# Reflective smartphone disengagement as a coping strategy against cyberbullying: A cross-country study with emerging adults from the United States and Indonesia

**DOI:** 10.1177/14614448241254015

**Published:** 2024-05-28

**Authors:** Maryam Khaleghipour, Kevin Koban, Anja Stevic, Jörg Matthes

**Affiliations:** University of Vienna, Austria; University of Vienna, Austria; University of Vienna, Austria; University of Vienna, Austria

**Keywords:** Cross-cultural, cyberbullying, gender, perceived stress, reflective smartphone disengagement, self-esteem

## Abstract

Cyberbullying is a highly prevalent phenomenon among emerging adults, and it may lead to severe psychosocial harm for some targets. Understanding how emerging adults can cope with cyberbullying by altering their media use but without risking one of their crucial social lifelines, mobile social media, during the process is essential. To this end, this study examines a stress-coping process that involves cyberbullying as a stressor and reflective smartphone disengagement as a well-balanced coping strategy, accounting for gender-related, dispositional, and cultural specificities of emerging adults (aged 16–25, *N* = 4029) from the United States and Indonesia. With substantial invariance across countries, findings show that cyberbullying is related to higher perceived stress, especially for men and people with high levels of self-esteem, which, then again, is associated with reflective smartphone disengagement, in particular among American men and people with higher self-esteem.

Many young people today live in hybrid realities (i.e. the weaving together of the offline and online worlds in a single holistic ecosystem; Granic et al., 2020) where social media can help them with seeking, maintaining, and further deepening peer relationships (Sutcliffe et al., 2023). However, social media use can also involve hostile interactions, among which *cyberbullying* may be particularly widespread among youth. Cyberbullying builds upon a traditional understanding of bullying that is typically defined as intentionally hurtful behavior by a person or group (i.e. *perpetrators*) repeatedly executed over time against someone (i.e. *targets*) who cannot easily defend themselves (Olweus, 1993). Cyberbullying shares these features but is situated online (Kowalski et al., 2014) and, thus, considered a permanent threat due to social media (Kowalski et al., 2020). Cyberbullying can take various forms, including exclusions from online activities, offensive or threatening messages, and distributing denigrating pictures or videos (Kowalski et al., 2014). Research estimates that between 10% and 50% of young people have been cyberbullied (Hinduja and Patchin, 2019), with emerging adults experiencing particularly high levels (Barlett and Chamberlin, 2017; Wang et al., 2019).

Cyberbullying is linked to psychological harm (Giumetti and Kowalski, 2022), which prompts targets to use coping strategies (Alipan et al., 2021; Orel et al., 2017). Coping strategies are defined as individual responses to stressors (Lazarus and Folkman, 1984), and cyberbullying is regarded as an intrusive stressor since targets cannot escape it without cutting an essential social lifeline: social media (see, for example, Nguyen, 2023). While *engaging* media has been emphasized as a coping strategy (Wolfers and Schneider, 2021), purposeful *disengagement* may also be suitable for balancing the benefits and harms of digital media (Vanden Abeele, 2021). Recently, Matthes et al. (2022) introduced the concept of *reflective smartphone disengagement* (RSD), defined as “individuals’ deliberate efforts to control and restrict smartphone use” (p. 1), as a compromise that may also be useful for coping with cyberbullying. However, RSD’s utility is still to be examined.

This study addresses this gap with a cross-country survey with emerging adults from the United States and Indonesia. First, conceptualizing and examining RSD as a behavioral emotion-focused avoidance coping strategy against cyberbullying victimization that focuses primarily on disengaging (rather than engaging) social media expands extant approaches on how social media use can result in beneficial coping outcomes and how detrimental outcomes may be avoided (Wolfers and Utz, 2022). Second, evidence shows that cyberbullying tends to be more consequential for women than men (Baldry et al., 2019), as well as for individuals with low self-esteem (Palermiti et al., 2017). Extending prior studies, we examine whether young people’s gender identification and self-esteem moderate the specified coping process. Third, given that most English-language publications on cyberbullying involve participants from North America, Europe, and Australia (Zych et al., 2015), we lack solid comparative evidence from other geographical contexts (Livingstone et al., 2016). Referring to indices by Hofstede Insights (2023), we address this shortage by investigating cyberbullying among emerging adults from the United States (i.e. a prototypically WEIRD (i.e. Western, Educated, Industrialized, Rich, and Democratic; Henrich et al., 2010) and individualistic country with relatively low power distance) and Indonesia (i.e. a prototypically collectivistic country from the global south with greater power distance). Given that cultural values such as individualism/collectivism and power distance have been identified as crucial components related to bullying (Smith and Robinson, 2019), our study, by examining two countries selected because they differ substantially across these parameters, contributes greatly to a more generalizable understanding of how young people experience and cope with cyberbullying.

## Theoretical framework

According to the transactional model of stress and coping (Lazarus, 2001), people are permanently confronted with situational demands that are evaluated for relevance and, if regarded relevant, weighted against self-perceived resources for coping with them. Once perceived coping demands outweigh one’s perceived current resources, stress is experienced together with a need to engage in coping strategies.

Lazarus and Folkman (1984) distinguished between problem-focused (i.e. solving the demands-resources imbalance when the stressful situation is perceived as controllable) and emotion-focused coping strategies (i.e. addressing undesired emotions raised by the demands-resources imbalance when the stressful situation is perceived as uncontrollable). Others, like Riva (2016), proposed multidimensional classifications, combining a cognitive-behavioral dimension (differentiating between coping with the demands-resources imbalance through thinking or behaving in a particular manner) and an approach-avoidance dimension (distinguishing between coping directed toward or away from the demands-resources imbalance to control a stressor or escaping from it, respectively).

As for media, a current review noted that social media fulfills different functions in this stress-coping process (Wolfers and Utz, 2022). Specifically, using social media can come with a diverse set of unique experiences (i.e. availability stress, approval anxiety, fear of missing out, connection overload, and online vigilance) that have been subsumed under the term digital stress (Hall et al., 2021). However, given that mobile social media use can provide connections to potentially supportive social contacts or communities, it may also allow access to resources for countering situational demands (Liu et al., 2016).

This study adopts this theoretical framework to study cyberbullying and RSD. Viewed through a transactional model lens, cyberbullying can be understood as a persistent stressor that may overwhelm available resources, increase stress, and, thus, necessitate coping (Raskauskas and Huynh, 2015). This stress-coping process may be strongly pronounced among emerging adults, given that this transitional period involves self-exploration and social status priorities amid coming-of-age instability (Arnett, 2007), and because they have been distinguished by previous research as prone to being targeted by cyberbullying (Barlett and Chamberlin, 2017; Wang et al., 2019).

Within this process, RSD can be conceptualized as a possible coping strategy. Generally, Wolfers and Schneider (2021) suggested thinking of media applications as tools that stand “orthogonal to coping strategies” (p. 13). Following this understanding, their review, as well as others (Wolfers and Utz, 2022), focused primarily on how people actively use media for different coping strategies. However, whether media is engaged in the first place or instead disengaged may be considered a coping strategy (rather than a coping tool) if media use is required for stressors to be salient. Given that widespread smartphone checking habits are known to be a gateway to media applications and content (Meier, 2022), including (socio-)environmental stressors like cyberbullying, disengaging smartphone use may be a proper target point.

In recent years, several disengagement strategies (see Radtke et al., 2022) have become popular beyond academia. Among these, RSD can be understood as intentional rule-setting for situationally appropriate smartphone use (rather than temporary abstinence), aiming to maximize benefits and minimize risks of mobile connectedness (Matthes et al., 2022). As such, it appears reasonable to theoretically integrate RSD into the transactional stress-coping process as a (primarily) *emotion-focused behavioral avoidance strategy* (expanding upon Matthes et al., 2023). Specifically, in the case of cyberbullying, targets may purposefully “engage” in RSD to situationally limit attention to what may be said about them online. This disengagement then inhibits unpleasant emotions emerging from becoming aware of being targeted again (and possibly even prevents cyberbullying from happening over time, similar to other strategies; Machackova et al., 2013).

Based on this reasoning, this study examines a stress-coping process that involves cyberbullying as a socio-environmental stressor, perceived stress as a manifestation of a demand-resource imbalance, and RSD as a coping strategy within a theoretically grounded mediation model, accounting for gender-related, dispositional, and cultural specificities.

### Cyberbullying victimization relates to perceived stress

Due to the rapid emergence of numerous platforms, social media has become the most common venue for cyberbullying (Giumetti and Kowalski, 2022). Although cyberbullying and traditional bullying share a definitional core (as both draw from Olweus, 1993), they still differ substantially. In contrast to traditional bullying, cyberbullying messages are more easily shared by perpetrators for de-contextualized online audiences, multiplying potential harm for targets, as they can be repeated constantly by others beyond their original lifetime (Livingstone et al., 2016). Moreover, online anonymity advantages perpetrators while making it more difficult for targets to respond effectively (Pabian and Vandebosch, 2021). Overall, longevity, context collapse, and anonymity can make cyberbullying even more stressful than traditional bullying (Sourander et al., 2010).

A wide range of negative consequences of cyberbullying has been studied, including feelings of sadness, depression, suicidal ideation (Giumetti and Kowalski, 2022), and, most crucially, a greater risk of self-harm and suicidal behaviors (Kowalski et al., 2014). Research further estimated that almost 30% of targets experience at least one symptom of severe stress (Nixon, 2014) resulting from unwilling exposure to negative and harmful comments, perceived isolation, or social exclusion (Kowalski et al., 2014). Thus, it appears plausible to understand cyberbullying as a socio-environmental stressor. We suggest,

*H1*. Cyberbullying will be positively associated with perceived stress.

### Perceived stress relates to reflective smartphone disengagement

From a transactional model of stress and coping perspective, a person copes with stressful situations through coping strategies. Naturally, each strategy comes with certain situational requirements to be effective and may lead to specific benefits. Problem-focused strategies typically require that the stressful situation is controllable and can lead to lasting resolution, while emotion-focused strategies can be used even under uncontrollable conditions where they can bring temporary relief (Carver et al., 1989). Approach strategies, however, can lead to quick problem-solving but can also result in distress, whereas avoidance strategies can reduce stress for a brief period but may also manifest long-term displeasure if applied alone (Roth and Cohen, 1986). Due to this functional complexity, people can find themselves searching for a fitting coping strategy.

Studies show that young targets often employ social media as a coping tool. Most notably, cybersecurity solutions (e.g. blocking or reporting) are frequently used for cyberbullying (Orel et al., 2017), even though these features may lose effectiveness if not followed by other strategies (Machackova et al., 2013). Moreover, Wolfers and Utz (2022) suggested that social media can be used for coping with stress by distracting from stress-inducing situations. In the case of cyberbullying, social media is typically where stressors are most present. Therefore, once targets use their smartphone to access social media, even for distraction, they run into the risk of being bullied (see Kowalski et al., 2014). Instead of engaging social media as a coping tool, purposefully disengaging it may help targets by limiting direct awareness and salience of cyberbullying and, thus, reducing unpleasant consequences (Machackova et al., 2013; Varela et al., 2022).

Bearing in mind young people’s preference to be connected and maintain social relationships through social media (Sutcliffe et al., 2023), RSD (Matthes et al., 2022) can be regarded as a moderately exclusionary emotion-focused behavioral avoidance coping strategy that emerging adults can apply flexibly without fully restricting their smartphone use when they experience cyberbullying-related stress. Similar to prior research documenting that great stress is linked to stronger engagement in self-protective behaviors such as avoiding social media altogether (Evelyn et al., 2022), it can, therefore, be assumed that emerging adults are more likely to consider RSD when stressed, with perceived stress as a mediator for links to cyberbullying victimization.

*H2*. Perceived stress will be positively associated with RSD.
*H3*. Perceived stress will mediate the relationship between cyberbullying and RSD.

### Gender identification and self-esteem as moderators

Research suggests that women are more susceptible to harmful consequences of social media use than men (Twenge and Farley, 2021). For one thing, women may experience more victimization because they tend to spend more time online (Craig et al., 2020). In addition, women typically focus more on being socially integrated, such that cyberbullying may more likely trigger social threat perceptions that can lead to internalized distress (Bandura, 1986).

Furthermore, women generally favor coping strategies that aim to decrease unpleasant emotions and help them avoid stressors (Panayiotou et al., 2017). In line with this, when women are under stress, they prefer emotion-focused coping strategies compared to problem-focused ones (Graves et al., 2021). Assuming that the same might be true for cyberbullying victimization, we hypothesize,

*H4*. Links between (1) cyberbullying and perceived stress, (2) perceived stress and RSD, and (3) cyberbullying and RSD will be stronger for self-identified women than men.

In addition to gender identification, self-esteem may also act as a moderator in the process of coping with cyberbullying. Earlier studies have revealed that low self-esteem can be a predictor of cyberbullying victimization and stronger negative consequences (Kowalski et al., 2012; Palermiti et al., 2017). Self-esteem is an important dispositional facilitator for stress resilience (Liu et al., 2021), such that individuals with high self-esteem typically engage in more effective coping, while those with low self-esteem often struggle with stressful situations because they lack coping resources (Orth et al., 2009). Based on this, it could be assumed that self-esteem weakens the stressful impact of cyberbullying. However, whether and how self-esteem moderates coping strategies is still unclear and has rarely been examined for cyberbullying victimization.

In relation to media coping, research has shown that people with high self-esteem often utilize mobile social media in a balanced manner, effectively coping with online stressors (Servidio et al., 2018). People with low self-esteem, however, have been linked to compulsive social media use and fear of missing out (Servidio, 2023), which might make them less likely to disengage their smartphones. Nevertheless, given that empirical insights into the moderating role of self-esteem are scarce, we ask,

*RQ1*. How will self-esteem moderate links between (1) cyberbullying and perceived stress, (2) perceived stress and RSD, and (3) cyberbullying and RSD?

### Cross-cultural differences

In general, cross-country studies avoid common fallacies, broadening our understanding beyond unrepresentative contexts (Papayiannis and Anastassiou-Hadjicharalambous, 2011). Specifically, the majority of cyberbullying work has focused on WEIRD populations (Zych et al., 2015), resulting in lacking evidence from other geographical and cultural contexts (Livingstone et al., 2016). WEIRD populations are by no means considered representative of other populations, particularly regarding behavioral tendencies (Henrich et al., 2010). Among factors for cross-national studies, cultural values have been researched extensively in relation to bullying (Smith and Robinson, 2019). Nevertheless, it is still unclear whether individuals across diverse young populations experience and cope with cyberbullying differently, despite its global prevalence. This study compares data from the United States and Indonesia, assuming that cultural differences (e.g. collectivism vs individualism, power distance, Hofstede Insights, 2023) may influence the outlined stress-coping process around cyberbullying and RSD. Thus far, cross-cultural studies on bullying have shown contradictory results. For example, Ji et al. (2016) argued that bullying is less common in collectivist societies, whereas Migliaccio and Raskauskas (2016) found the opposite. Aside from incidences, cultural aspects may manifest in different reactions and coping tendencies (Erişti, 2019), particularly given higher overall social media use in Indonesia (Howarth, 2023) and higher prevalences of social media addiction in collectivist nations (Cheng et al., 2021), which both may make disconnection more difficult (see Nguyen, 2023).

Furthermore, gender identification and self-esteem may also have varying effects on how targets deal with cyberbullying across countries. In this regard, Bleidorn et al. (2016) found that greater power distance is related to smaller gender gaps, while individualism is associated with larger gender differences. Compared to Indonesia, gender identification may thus have a stronger impact on young people in the United States due to lower scores on power distance and higher scores on individualism (see Hofstede Insights, 2023). In addition, Asian people often report lower self-esteem than American people (Lyu et al., 2019), which may influence whether buffer mechanisms emerge. Yet, there is a dearth of research on how gender identification and personality traits moderate coping, particularly with cyberbullying, which complicates formulating hypotheses. Instead, we ask,

*RQ2*. How do relationships between (1) cyberbullying and perceived stress, (2) perceived stress and RSD, and (3) cyberbullying and RSD differ between samples from the United States and Indonesia?
*RQ3*. How do the moderating effects of gender identification and self-esteem differ between samples from the United States and Indonesia?

## Method

Supplemental Material, including anonymized data, R analysis script, and detailed analysis output files are available at https://osf.io/764zg/.

### Participants

We conducted a large-scale cross-national survey in the United States (collected in August 2022) and Indonesia (collected in December 2022) as part of a larger project that examines links between social media use and well-being among emerging adults (i.e. 16- to 25-year-olds). The Institutional Review Board of the Department of Communication at the University of Vienna declared the study to be of minimum ethical risk (IDs: IRB_COM 20220723_035 and IRB_COM 202001118_063). US participants were recruited by *Kantar*, and Indonesian participants by *Dynata*, both global market research companies with large and permanently monitored pools of young participants available across the world for taking part in online surveys. Participants were included if they (1) provided active informed consent, (2) were between 16 and 25 years old, (3) were from the United States or Indonesia and proficient in English or Indonesian, respectively, and (4) were active social media users. Sampling was set up in both countries toward balanced distributions of gender identification (i.e. half female, half male) and age (i.e. half being 16–20, half being 21–25 years old). Participants who completed the survey were excluded post hoc if they (1) failed attention checks or (2) were flagged as speeders (based on criteria by Greszki et al., 2014). A total of *N* = 4029 emerging adults (*n* = 2044, 50.7% men, *n* = 1985, 49.3% women; *M_age_* = 21.07, *SD_age_* = 2.45) remained in the final sample. Frequency distributions across both countries was: The United States: *n* = 1922 [47.7%] with *n* = 985 [51.3%] male and *n* = 937 [48.8%] female identification, mean age of 21.26 years (*SD* = 2.52), and *n* = 1316 [69.3%] without and *n* = 584 [30.7%] with post-secondary education; Indonesia: *n* = 2107 [52.3%] with *n* = 1059 [50.3%] male and *n* = 1048 [49.7%] female identification, mean age of 20.89 years (*SD* = 2.37), and *n* = 1421 [67.8%] without and *n* = 675 [32.2%] with post-secondary education (see Supplemental Material for more demographic information).

Checking for cross-country differences, results revealed a significant but very small difference for age, *t*(3934.2) = 4.80, *p* < .001, *Cohen*’*s d* = .15, showing that the participants from the United States were slightly older than the participants in Indonesia. Significant differences were also found for perceived stress, *t*(3749.3) = –3.71, *p* < .001, *d* = –.12; RSD, *t*(3816.8) = –19.57, *p* < .001, *d* = –.62; and self-esteem, *t*(3737.7) = –9.18, *p* < .001, *d* = –.29. Indonesian participants reported higher stress (The United States: *M* = 3.28, *SD* = 1.08 vs Indonesia: *M* = 3.39, *SD* = 0.90), RSD (The United States: *M* = 3.25, *SD* = 0.90 vs Indonesia: *M* = 3.77, *SD* = 0.78), and self-esteem (The United States: *M* = 3.14, *SD* = 1.08 vs Indonesia: *M* = 3.43, *SD* = 0.89).

### Measures

The initial survey was constructed in English before we applied a translation backward-translation process for the Indonesian survey. Here, a native Indonesian and proficient English speaker blind to the research first translated all English items. We then ensured that the translation was adequate through another native Indonesian and proficient English speaker blind to the research purpose who translated the Indonesian items back into English. Afterward, the second and third authors discussed the back-translation with both translators to determine whether items were accurately translated and understood. Notably, additional analyses including confirmatory factor analysis and measurement invariance testing were conducted across countries (demonstrating good-to-acceptable fits and partial metric invariance) and are reported in the Supplemental Material.

#### Cyberbullying

To gauge cyberbullying, we used four items from Stewart et al. (2014). On a 5-point Likert-type scale (1 = never, 5 = always), we asked participants whether they experienced situations describing typical cyberbullying behaviors ( e.g. “someone told lies about you to make others not like you anymore” or “someone put you down by posting cruel gossip, rumors, or something else hurtful”) during the last for weeks (The United States: *M* = 2.26, *SD* = 1.20, α = .91; Indonesia: *M* = 2.20, *SD* = 1.09, α = .91).

#### Perceived stress

We measured perceived stress with six items from Cohen et al. (1983). On a 5-point Likert-type scale (1 = never, 5 = very often), participants were asked how often they had several stressful situations (e.g. “been upset because of something that happened unexpectedly” or “felt nervous and stressed”) in the past 4 weeks (The United States: *M* = 3.28, *SD* = 1.08, α = .91; Indonesia: *M* = 3.39, *SD* = 0.90, α = .87).

#### Reflective smartphone disengagement

We used six items from Matthes et al. (2022). Participants indicated their agreement (1 = I strongly disagree, 5 = I strongly agree) with statements such as “There are situations in which I do not want to be reachable, which is why I switch off the mobile phone, intentionally put it away, or do not look at it” (The United States: *M* = 3.25, *SD* = 0.90, α = .79; Indonesia: *M* = 3.77, *SD* = 0.78, α = .77).

#### Moderating variables

**Gender identification.** We asked participants which gender describes their identity best via a closed-ended item. To match both samples and since *N* = 68 in the US sample does not allow for meaningful findings, non-binary identifying participants were excluded. Accordingly, gender identification was binary coded (1 = male; 2 = female).

**Self-esteem.** Self-esteem was assessed with four items from the need satisfaction scale (Jamieson et al., 2010). On a 5-point Likert-type scale (1 = not at all, 5 = entirely), participants were instructed to think about their social media use in the past 4 weeks and indicate how they felt regarding their social media connections (e.g. “I felt good about myself” and “I felt secure”; The United States: *M* = 3.14, *SD* = 1.08, α = .87; Indonesia: *M* = 3.43, *SD* = 0.89, α = .82).

## Statistical analysis

We ran moderated mediation models for each sample, entering cyberbullying victimization as independent variable, perceived stress as mediator, and RSD as dependent variable. Gender identification and self-esteem were involved (1) as correlates of perceived stress and RSD and (2) through interaction terms with cyberbullying (predicting perceived stress and RSD, respectively) and perceived stress (predicting RSD). To examine country-level moderations, we specified another set of mediated moderation models where we conducted multigroup analyses, comparing iteratively whether fit differs significantly between models with freely estimated coefficients and models where coefficients were constrained to be equal across groups (Hayes et al., 2013). Age and education were covariates. Cyberbullying, perceived stress, and self-esteem were mean-centered. Listwise exclusion was conducted for missing data (*n* = 29 in the US sample [1.51%] and *n* = 11 in the Indonesian sample [0.52%]); however, robustness checks suggested little impact (see Supplemental Material).

## Results

Main results (including standardized coefficients) are displayed in Table 1.

**Table 1.** Overview of the moderated mediation analyses.

	US sample (*N* = 1922)		Indonesian sample (*N* = 2107)	
	Perceived stress *b* (*SE*), β	Reflective smartphone disengagement *b* (*SE*), β	Perceived stress *b* (*SE*), β	Reflective smartphone disengagement *b* (*SE*), β
Cyberbullying	.38 (.07), .42***	**.20 (.06), -27** ***	.30 (.06), .36, ***	**.02 (.05), .02**
Self-esteem	**-.16 (.03), -.16** ***	**.14 (.02), .17** ***	**-.26 (.02), -.26** ***	**.23 (.02), .26** ***
Cyberbullying ⨯ self-esteem	.13 (.02), .17***	.05 (.02), .09**	.11 (.02), .13***	.03 (.02), .04
Gender identification	.33 (.05), .15***	.10 (.04), .05*	.34 (.04), .19***	.12 (.03), .08***
Cyberbullying ⨯ gender identification	-.12 (.04), -.21**	-.08 (.04), -.16*	-.06 (.04), -.12	-.01 (.03), -.02
Education	-.09 (.05),- .04	-.02 (.05), -.01	-.004 (.05), -.002	-.01 (.04), -.01
Age	-.003 (.01), -.01	.02 (.01), -05*	-.03 (.01), -.07**	.02 (.01), .06*
Perceived stress		.23 (.07), .27**		.15 (.07), .17*
Perceived stress ⨯ self-esteem		-.05 (.02), -.07*		-.07 (.02), -.08**
Perceived stress ⨯ gender identification		-.05 (.05), -.09		-.01 (.04), -.01
*R* ^2^	13.5%	14.0%	18.9%	8.9%

Gender identification: 1 = male; 2 = female. Education: 1 = only secondary; 2 = post-secondary education. Bold indicates significant multigroup comparisons.

* *p* < .05; **p < .01; ***p < .001.

### US sample

Concerning perceived stress, the analysis revealed a significant association with cyberbullying victimization, *b* = .38, *SE* = .07, *p* < .001, supporting H1. This relationship was moderated by self-esteem, *b* = .13, *SE* = .02, *p* < .001, and gender identification, *b* = –.12, *SE* = .04, *p* = .006. In other words, the association between being cyberbullied and perceived stress in the American sample increased with higher levels of self-esteem (answering RQ1a) and was more positive among young males compared to female participants (contradicting H4a). Both self-esteem, *b* = –.16, *SE* = .03, *p* < .001, and gender identification, *b* = .33, *SE* = .05, *p* < .001, emerged as significant, while neither education, *b* = –.09, *SE* = .05, *p* = .085, nor age did so, *b* = –.003, *SE* = .01, *p* = .801. Accordingly, lower self-esteem and self-identification as female were associated with higher levels of perceived stress (when cyberbullying is average).

With regard to RSD, cyberbullying victimization did emerge as significant, *b* = .20, *SE* = .06, *p* < .001. Furthermore, this association was moderated by both self-esteem, *b* = .05, *SE* = .02, *p* = .001, and gender identification, *b* = -.08, *SE* = .04, *p* = .031. Again, these results point toward an increasingly positive link between cyberbullying and RSD for higher self-esteem scores (answering RQ1b) as well as a stronger positive relationship among male—compared to female-identifying participants (oppositional to H4b).

In line with H2, perceived stress was also found with a significant link to RSD, *b* = .23, *SE* = .07, *p* = .001. In addition, self-esteem, *b* = –.05, *SE* = .02, *p* = .017, but not gender identification, *b* = –.05, *SE* = .05, *p* = .273, moderated this association. In other words, the association between perceived stress and RSD was less positive once participants scored higher on self-esteem (answering RQ1c), whereas no such variance could be identified across gender identifications (no support for H4c). Higher self-esteem, *b* = .14, *SE* = .02, *p* < .001, female gender, *b* = .10, *SE* = .04, *p* = .017, and being older, *b* = .02, *SE* = .01, *p* = .023, were related to greater RSD (when stress is average). Education was not significant, *b* = –.02, *SE* = .05, *p* = .643.

Finally, concerning a possible mediation of cyberbullying victimization on RSD through perceived stress (using 5000 bootstrap confidence intervals), we found that the indirect effect is different from zero, 95% CI = [0.03, 0.17], qualifying a significant total effect, 95% CI = [0.17, 0.41]. H3 was supported.

### Indonesian sample

Our analysis again demonstrated a significant positive relationship between cyberbullying victimization and perceived stress, *b* = .30, *SE* = .06, *p* < .001. This result again supports H1. In contrast to the US sample, however, only the interaction term with self-esteem qualified this relationship, *b* = .11, *SE* = .02, *p* < .001, not with gender identification, *b* = -.06, *SE* = .04, *p* = .083. According to these results, the association between being cyberbullied and perceived stress became more positive with greater self-esteem, which answers RQ1a. Conversely, no variability in slopes was found across gender identifications. Accordingly, no support was found for H4a. Similar to Americans, participants with lower self-esteem, *b* = –.26, *SE* = .02, *p* < .001, and female gender, *b* = .34, *SE* = .04, *p* < .001, experienced higher stress levels (when cyberbullying is average). Again, education was not significant, *b* = -.004, *SE* = .05, *p* = .938; however, younger participants reported significantly more stress experiences, *b* = –.03, *SE* = .01, *p* = .003.

Concerning RSD, cyberbullying was not significant, *b* = .02, *SE* = .05, *p* = .741. Neither self-esteem, *b* = .03, *SE* = .02, *p* = .160, nor gender identification, *b* = –.01, *SE* = .03, *p* = .837, moderated this (non-)relationship. Said differently: Cyberbullying victimization was not related to RSD, and this (non-)relationship did not vary across different self-esteem levels or gender identifications (answering RQ1b and not supporting H4b).

Perceived stress, however, came up as significant, *b* = .15, *SE* = .07, *p* = .039, supporting H2. This association was also significantly moderated by self-esteem, *b* = –.07, *SE* = .02, *p* = .004, but not gender identification, *b* = -.01, *SE* = .04, *p* = .872. Similar to the American sample, the link between perceived stress and RSD got weaker with increasing self-esteem (answering RQ1c), while no such was found across gender identifications (no supporting H4c). Furthermore, higher self-esteem, *b* = .23, *SE* = .02, *p* < .001, and female gender identification, *b* = .12, *SE* = .03, *p* < .001, were related to greater RSD (when stress is average), just as was older age, *b* = .02, *SE* = .01, *p* = .016. Varying education, *b* = –.01, *SE* = .04, *p* = .793, resulted in no significant differences.

Finally, using 5000 bootstrap replications for estimating confidence intervals, we again found that the indirect effect of cyberbullying on RSD through perceived stress is different from zero, 95% CI = [0.01, 0.10], despite a non-significant total effect, 95% CI = [–0.04, 0.17]. H3 was again supported.

### Multigroup analyses

As a formal test of moderation by country, we examined whether constraining each pair of paths to be equal across groups results in a worse fit compared to the freely estimating model (Hayes et al., 2013). These model comparisons turned out significant for the following three paths: (1) self-esteem predicting perceived stress, the United States: *b* = –.16, *SD* = .03 vs Indonesia: *b* = –.26, *SD* = .02; Δχ²(1) = 10.89, *p* = .001, (2) cyberbullying predicting RSD, the United States: *b* = .20, *SD* = .06 vs Indonesia: *b* = .02, *SD* = .05; Δχ²(1) = 6.24, *p* = .012, and (3) self-esteem predicting RSD, the United States: *b* = .14, *SD* = .02 vs Indonesia: *b* = .23, *SD* = .02; Δχ²(1) = 9.16, *p* = .002. For the other paths, no significant decrease in fit was found. Accordingly, answering RQ2 and RQ3, our data provide support for a meaningfully diverging association between cyberbullying and RSD, with stronger cyberbullying victimization coming with greater RSD among emerging adults from the United States whereas no such link was found among Indonesian emerging adults. Moderations with self-esteem and gender did not differ meaningfully.

## Discussion

Cyberbullying is a highly prevalent issue among youngsters from all over the world that can come with severe psychosocial harm (Kowalski et al., 2014). Since permanently leaving online spaces like social media altogether may not be feasible given its great importance for young people’s social connectedness (Sutcliffe et al., 2023), we argued that RSD (Matthes et al., 2022) may present a workable coping strategy for dealing with cyberbullying victimization.

Taking the cross-country perspective with two samples from the United States and Indonesia, we found a strong positive relationship between cyberbullying victimization and perceived stress that aligns with previous research (Nixon, 2014), highlighting that cyberbullying targets feel highly stressed about it regardless of the culture they live in. Importantly, we assumed that when targets feel stress due to cyberbullying victimization, they may consider RSD as a useful coping strategy. Results supported this assumption in both samples, showing not only that perceived stress is significantly correlated with RSD but also that there might be an indirect effect between cyberbullying victimization and RSD through perceived stress. Accordingly, targets may try to cope with stressful cyberbullying by applying RSD as a (primarily) emotion-focused behavioral avoidance strategy. Purposefully disengaging from one’s smartphone (which also implies disengagement from social media as the main source of cyberbullying; Evelyn et al., 2022) may benefit targets by limiting their momentary awareness of cyberbullying taking place and, thus, reducing victimization experiences (Machackova et al., 2013; Varela et al., 2022). While we acknowledge that such coping could lose its effectiveness if not followed by coping that helps tackle cyberbullying (problem-focused/approach coping strategies; Alipan et al., 2021), it needs to be noted that RSD might be particularly precious as it allows for an urgent breather after which targets may be more capable to actively engage the problem at hand. Our study gives a first indication toward RSD as a coping strategy that may not be limited to specific cultures.

As person-level moderators, we examined gender identification and self-esteem. For gender identification, we expected stronger adverse effects of cyberbullying on stress for women and a stronger relationship between stress and RSD as a coping strategy for women. Against our expectations, and only in the US sample, our results revealed that the relationship between cyberbullying and perceived stress is more pronounced among men. A possible reason for this finding could be that male emerging adults from the United States rarely report cyberbullying to others who may help them with it, while female compatriots talk more openly about it with friends and adults (Li, 2006). That is, men from an individualist country like the United States may be more likely to deal with cyberbullying on their own, which could result in more intense stress (Kowalski et al., 2012). However, future research is needed to investigate the underlying factors contributing to this unexpected result, refining this interpretation, for instance, by accounting for how differently both cyberbullying and stress can manifest themselves and are experienced across genders over the course of adolescence and emerging adulthood (Smahel et al., 2020). Similarly, the link between cyberbullying and RSD, an indication of individuals’ likelihood to consider disengaging their smartphone when being cyberbullied, was also found more positive for young US men. Distancing temporarily from the most relevant source of their digital stress may be considered a more viable option for young men because they tend to have less tight online networks or more strongly seek offline social support. Notably, no gender identification moderations were found in Indonesian emerging adults, suggesting these preferences may vary across cultural contexts and should, therefore, be considered in a more focused way in future studies.

In addition to gender identification, self-esteem turned up as a relevant positive moderator for the relationships between cyberbullying and stress in both samples such that they became stronger for participants who scored higher on self-esteem. Somewhat counterintuitively, this finding suggests that experiencing harmful stressors like cyberbullying might be especially hard for young people with relatively high self-regard who, generally, tend to experience less stress and, thus, may be expected to be more resilient. One explanation could be that individuals with high self-esteem expect less negative feedback than those with low self-esteem, and losing support from peers undermines their sense of self-worth. The stress-RSD relationship, on the other hand, was found weaker for those higher in self-esteem across both countries, suggesting that a positive self-evaluation makes them less likely to engage in RSD. Previous research has indicated that individuals with high self-esteem often do not engage in emotion-based coping, possibly because their robustly positive self-regard is more sufficiently able to protect them from short-term consequences (Liu et al., 2021; Orth et al., 2009). By implication, RSD may simply be a less relevant option for youth with high self-esteem.

Regarding cultural context, we found differences only concerning the direct relationships between cyberbullying and RSD. That is, emerging adults from the United States were more likely to disengage their smartphones in a reflective manner than young people from Indonesia when experiencing cyberbullying victimization. Generally, given that social media plays a greater everyday-life role among Indonesians compared to American youngsters (as evidenced by overall use; Howarth, 2023), it could be less feasible for emerging adults from Indonesia to disengage their smartphones, even in the case of being constantly confronted with cyberbullying through it, making this coping strategy somewhat impractical. Furthermore, accounting for the difference between individualistic and collectivistic cultures (Hofstede Insights, 2023), it could also be that emerging adults from the United States just do not prioritize seeking or depending on online connections to the same extent as those in Indonesia and prefer the avoidance and detachment coping style (see Hu et al., 2018). Alternatively, the other way around, it could be more challenging for Indonesian emerging adults because coping through online social connections might be a viable option for managing cyberbullying due to more prevalent collectivism.

### Limitations

Several limitations need to be acknowledged. First, this study is cross-sectional, which does not allow for causal inferences. Our findings represent first evidence concerning whether RSD may be linked to cyberbullying victimization and stress. As a next step, future studies should aim to confirm the theory-based directionality by means of longitudinal studies, which may also shed light on the effectiveness of RSD as a coping strategy. Second, our data are based on self-reports, which are vulnerable to various biases. Third, while we measured how frequently participants experienced cyberbullying, we did not operationalize how cyberbullying unfolded. Follow-up research could account for cyberbullying content and employ more fine-grained measures to capture varying experiences across different contexts to examine content-tailored copings. Fourth, our study is limited to male and female emerging adults and may not fully represent other age groups or other gender identities, as experiences and coping strategies with cyberbullying may vary across different gender identities and age cohorts (Smahel et al., 2020). Fifth, our comparative findings are limited to emerging adults across the United States and Indonesia, which may not translate straightforwardly to other primarily individualist or collectivist countries. Conducting more culturally sensitive investigations in the future would enhance our understanding of how cultural factors affect the variables being studied. Finally, while using professional market research companies is typically considered superior to other recruitment means when attempting to sample diverse participants across different countries (e.g. crowdsourcing platforms; see Moss et al., 2023), it still needs to be acknowledged that they also may contain certain biases, for instance, concerning who is registered in their participant pools and who not.

## Conclusion

Our cross-country study makes a significant contribution to the existing theory by conceptualizing and integrating RSD as a behavioral emotion-focused avoidance coping strategy for cyberbullying victimization into an overarching transactional stress-coping framework. Our findings indicate that cyberbullying victimization may prompt disengagement from smartphones as a coping strategy which stands in stark contrast to widespread recommendations to actively engage with victimization experiences. Accounting for gender-related, dispositional, and cultural specificities, our analysis reveals that this stress-coping process varies across cultures, genders, and traits, with the noteworthy exception that RSD may be considered a more suitable coping strategy among American youngsters. Bearing in mind the notorious lack of cross-cultural investigations across WEIRD and non-WEIRD contexts, our study offers valuable contributions, particularly with respect to a cross-cultural understanding of media coping for an all-too-common experience among youth.
